# Alterations in gut microbiota caused by major depressive disorder or a low FODMAP diet and where they overlap

**DOI:** 10.3389/fnut.2023.1303405

**Published:** 2024-01-08

**Authors:** Simone O’Neill, Michelle Minehan, Catherine R. Knight-Agarwal, David B. Pyne

**Affiliations:** ^1^University of Canberra Research Institute for Sport and Exercise, Faculty of Health, University of Canberra, Canberra, ACT, Australia; ^2^Faculty of Health, University of Canberra, Canberra, ACT, Australia

**Keywords:** microbiota, gut-brain-axis, major depressive disorder (MDD), mental health, dietary intervention, macronutrients, FODMAP diet

## Abstract

Beneficial changes in microbiota observed in individuals with a major depressive disorder (MDD) may be initiated with a low fermentable oligosaccharide, disaccharide, monosaccharide, and polyol (FODMAP) elimination diet. Academic Search Ultimate, APA PsychINFO, Cochrane Library, MEDLINE, Scopus and Web of Science were searched for original research documenting differences in microbiota in MDD or changes with a low FODMAP diet in adults (age 18 years +). Studies with fecal microbiota, 16 s RNA sequencing and QIIME pipelines were included. Studies using antibiotics, probiotics, and medications such as antidepressants were excluded. Additionally, studies based on a single gender were excluded as gender impacts microbiota changes in MDD. Four studies addressed differences in microbiota with MDD and another four assessed shifts occurring with a low FODMAP diet. The abundance of *Bacteroidetes*, *Bacteroidaceae* and *Bacteroides* were lower in individuals with MDD but increased with a low FODMAP diet. Abundance of *Ruminoccaceae* was lower and *Bilophila* was higher with both a low FODMAP diet and MDD. These results provide preliminary evidence that a low FODMAP diet might drive changes in microbiota that also benefit people with MDD. Further research to assess whether a low FODMAP diet can treat MDD through modification of targeted microbiota is warranted.

## 1 Introduction

Depression is a mood disorder with numerous multifactorial consequences at individual and community levels. The biological factors believed to influence depression include oxidative stress, inflammation, increased cortisol production, decreased levels of brain-derived neurotrophic factor (BDNF), impaired mitochondrial ATP production, and microbiota through shifts in the metabolites released (1). Lifestyle interventions can modify these pathways (2, 3) potentially offering alternative treatment modalities.

Until recently, treatment of depression was limited to pharmacological and psychosocial methods (4) with varying degrees of success (5, 6). Additionally, concerns have been raised that some pharmacological interventions are possibly unsafe, particularly with long term use (7). Consequently, these challenges have led to renewed interest in potential lifestyle interventions, such as diet and exercise, alongside more traditional treatments (8, 9). Investigations into diet and depression have largely relied on epidemiological studies, with evidence of an inverse relationship between diet quality and depression (10, 11). Only a small number of studies have directly examined the effect of whole dietary interventions on depression (12–15). These indicate dietary change can have a beneficial impact, but have primarily focused on a Mediterranean-style diet (16). Investigating a wider range of diet types may identify alternatives to improve outcomes for people living with a major depressive disorder (MDD).

The microbiome impacts depression through production of metabolites and regulation of neurotransmitters (17). One recent review of 24 studies reported microbiota shifts in individuals living with MDD compared to healthy controls, with 87% of the studies reporting differences in β-diversity (18). Taxa positively associated with MDD symptom severity included *Blautia* (27% studies), *Parabacteroides* (18% studies) and *Ruminococcus* (18% studies), while *Faecalibacterium* (36% studies), *Roseburia* (18% studies) and *Veillonella* (18% studies) were inversely associated. Diet is well established as one of many factors that can influence the microbiota (19). A low fermentable, oligosaccharide, disaccharide, monosaccharide, and polyol (FODMAP) diet can change the microbiota, through reduction of available fermentable short chain carbohydrates (20) which may offer a potential dietary intervention for management of MDD. Two studies that evaluated changes in irritable bowel syndrome symptoms, such as abdominal pain, bloating and stool consistency, with a low FODMAP diet coincidentally reported small reductions in depression (21, 22), however neither study measured alterations in the microbiota.

Further research is needed to investigate how diet can be used to modulate mood through the gut microbiota (23). Currently, no studies have evaluated the effect of a low FODMAP diet on microbiota and MDD. This review aimed to evaluate the potential for a low FODMAP diet to change the microbiota in a way that may benefit people with MDD. A low FODMAP diet could be a viable dietary intervention for MDD by beneficially modifying microbiota.

### 1.1 Links between the gut microbiota and major depressive disorder

The pathophysiology of depression is linked to four primary interacting pathways: brain dysfunction, the hypothalamus-pituitary–adrenal (HPA) axis, the immune system, and the gut brain axis (24). The gut-brain axis is important for healthy brain function, with dysregulation of the gut microbiota associated with mood disorders (25, 26). The gut brain, along with the nervous system, HPA axis and immune system, can influence other organs to regulate both the brain and behavior (24). The proposed mechanism is through production of metabolites, particularly short chain fatty acids (SCFA) which are used to send signals to the brain via the blood stream (27). Studies of SCFA production in MDD patients reveal a reduction in butyrate and acetate producers (25, 27). Moreover, SCFAs can cross the blood brain barrier (BBB) and have a role in maintaining its integrity (28).

Psychological stress has been hypothesized to impair the nervous system, HPA axis and immune system leading to depression (24). For example, immune dysregulation with chronic inflammation is thought to induce neuroinflammation (29). A reduction in butyrate producers may further compound this with butyrate having anti-inflammatory properties (27). Regulating gut microbiota can improve brain dysfunction and abnormalities of the immune system and HPA axis (24). Importantly, restoration of the gut microbiota using pre-and pro-biotics, healthy diet or fecal microbiota transplantation can elicit improvements in MDD (26). However, the complexity of the gut microbiota makes it challenging to identify causation (25). Many factors, such as gender (30) and age (31), appear to influence the microbiota composition in MDD. Increased relative abundance of *Actinobacteria* in female MDD patients compared to healthy female controls and decreased relative abundance of *Bacteroidetes* in male MDD patients compared to healthy male controls, has been reported by Chen et al. (30). Variations with age have also been identified with young MDD patients having increased *Prevotellaceae*, *Veillonellaceae*, *Acidaminococcaceae* compared with middle aged MDD patients who have increased *Lachnospiraceae*, *Ruminococcaceae* and *Peptostreptococcaceae* (31). Clarifying the influence of the microbiota will inform rebalancing of the microbiota through lifestyle interventions and alleviate the impact of psychological stress and disease on the nervous and immune systems.

### 1.2 Links between the gut microbiota and diet

Diet is one of several factors that influences the composition of the gut microbiota (19). Complex diets provide a range of substrates for growth-promoting and-inhibiting factors, with alterations in diet yielding compositional changes in the microbiome within 24 h (32). These changes can promote healthy shifts in the microbiota-gut-brain axis (23). Shifts in microbiota composition have been quantified in a range of cohorts including healthy adults following Mediterranean (33), vegetarian, vegan, and omnivorous diets (34, 35). Vegetarian diets yielded a lower ratio of *Clostridium* cluster XIVa (34, 35) and higher ratios of *Bacteroides thetaiotaomicron and Clostridium clostirdioforme* than an omnivorous diet, and *Faecalibacterium prausnitzii* was higher in a vegan diet (34). However, no specific food or food group has been identified that directly causes these changes (35).

The impact of protein on the microbiota is dependent on protein type (36, 37). Animal protein is associated with increased branch chain fatty acids (BCFA) (19, 37) and decreased SCFAs (36) as by-products of protein fermentation. Bile tolerant anaerobes such as *Bacteroides, Alistipes* and *Bilophila* increase in prevalence with intake of animal protein, while levels of *Firmicutes*, that promote digestion of plant polysaccharides, decrease (32). Ingestion of whey retentate reduced abundance of the pathogenic *Bacteriodes fragilis* and *Clostridium perfringens* (38). These changes likely increase the risk of negative health outcomes such as cardiovascular disease and irritable bowel disease (IBD) (37). Plant-based sources of protein, such as pea protein, can elicit an increase in *Bifidobacterium* and *Lactobacillus Enterococcus* (39). Plant-based proteins show increased SCFA production which is understood to exert anti-inflammatory effects (40). Overall impact of dietary protein on the gut microbiota is highly variable dependent upon the source which has implications in the design of dietary interventions for diseases.

Fat intake shifts the microbiota composition through alterations in bile acid composition and secretion (19). Total dietary fat intake is inversely associated with *Prevotella* incidence, and a high fat diet (~ > 35% of total energy intake) can increase *Clostridales, Bacteroides, Bilophila* and *Faecalibacterium prausnitzii* (36, 41). In contrast, a low-fat diet (~ < 30% of total energy intake) can yield an increase in *Bifidobacterium* (41). However, both the quantity and quality of the fats influence the outcome (37). The abundance of *Firmicutes*, *Proteobacteria* (42), *Bacteroides* (36) *Faecalibacterium prausnitzii* (36, 41), and *Bilophila* (36) increases, and *Bacillus bifidus* and *Bacteroidetes* (37) decreases, with high saturated fat diets (~20% saturated fat of total energy). Increased saturated fat intake is associated with increased incidence of sulfate reducing bacteria which can adversely affect the gut mucosa increasing inflammation and IBD risk (37). High unsaturated fat intake can increase *Bifidobacterium* (41) and *Akkermansia muciniphila* (36). Unsaturated fats can be further divided into monounsaturated (MUFA) and polyunsaturated (PUFA) types based on their chemical structures. Increased intake of MUFA has no impact on the diversity of the microbiota but correlates positively with *Parabacteroides, Prevotella, Turicibacter* genra and the *Enterobacteriacea* family. These fats are also associated with a decrease in the prevalence of *Bifidobacterium* (37) and decreased bacterial numbers compared with high carbohydrate and high glycemic index diets (41). PUFA intake can restore the ratio between *Firmicutes/Bacteroidetes* and increase the incidence of anti-inflammatory SCFAs such as butyrate (37). Therefore, dietary interventions aimed at moderating the microbiota for disease should consider both quantity and sources of dietary fats.

Total carbohydrate intake is the largest dietary predictor of microbiota diversity (43). The traditional Western diet with its emphasis on refined grains, starch and added sugars can impact negatively on the microbiota. Carbohydrates can be classified as either digestible or indigestible (44). The former includes glucose, fructose, sucrose and lactose, which are broken down in the small intestine by enzymes and released into the bloodstream increasing insulin (36). A diet high in glucose, fructose and sucrose can increase *Bifidobacteria* and decrease *Bacteroides* abundance (45). A decrease in the abundance of *Clostridia* cluster XIVa occurs with increased lactose intake, and supplementation with lactose increased the fecal concentration of beneficial SCFAs (36). Digestible carbohydrates can modulate microbiota diversity, which needs to be considered when developing dietary interventions promoting improvement in disease outcomes.

Indigestible carbohydrates, also known as fiber, reach the large intestine without being broken down by human digestive processes (44). Here the microbiota ferments them with products such as SCFAs being produced (36). Overall, a decrease in fiber intake results in decreased SCFA production with butyrate producers, including *Roseburia, Eubacterium rectale*, and *Faecalibacterium prausnitzii* being impacted (19). The prevalence of *Bifidobacteria* and *Lactobacilli* can increase with fiber intake (19). Similar to both proteins and fats, the type and quantity of dietary fiber also influences outcomes (37). Inulin intake, for example, is linked to increases in *Bifidobacterium* (2.8 fold), *Lactobacilli-enterococci* (2.4-fold) and decreases in *Clostridium* spp. (1.1-fold) (46, 47). Whereas intake of resistant starch increases abundance of *Bifidobacterium* (5.9-fold), *Roseburia*, *Rumminococcus* (4.5-fold), *Oscillibacter*, *Eubacterium rectale*, *Actinobacteria* (3.7-fold), *Bacteroidetes* (1.2-fold increase), *Ruminococcaceae* (1.3-fold), *Bifidobacteriaceae* (5.3-fold), *Porphyromonadaceae* (5.7-fold), and *Parabacteroides* (3.8-fold), and decreased *Firmicutes* (0.97-fold) (48, 49). Carbohydrate intake is easily modifiable and may be profoundly influential on microbiota composition and therefore a priority for manipulation to change treatment outcomes.

## 2 Methods

The clinical studies assessed here and outlined in Table 1 were sourced through searches of Academic Search Ultimate, APA PsychINFO, Cochrane Library, MEDLINE, Scopus, and Web of Science from inception to September 2023 employing terms “microbiome”, “depression” or low “FODMAP” diet and their associated medical subject headings (MeSH). Database searches identified 3,924 articles addressing differences in microbiota with depression and 1,405 studies investigating shifts in the microbiota with a low FODMAP diet. The resulting articles were manually searched to identify literature that addressed fecal microbiota in adult populations, with either MDD compared to healthy controls, or consumption of a low FODMAP diet compared to either a high FODMAP or habitual diet. Studies involving use of medications and probiotics or focused on single genders were excluded. Outcomes were measured using 16 s rRNA sequencing and bioanalytic analysis was completed using QIIME pipelines. To limit the impact of different pipelines on microbiota analysis only QIIME pipelines were included (58). This selection was due to its use in the only four studies investigating shifts in the microbiota with a low FODMAP diet. Table 1 presents details of the selected studies.

**Table 1:** Summary of human studies investigating the alterations in abundance of microbiota in major depressive disorder (MDD) and a low FODMAP.

References Study Design database	Aim of study	Technique	Cohort	Outcomes
Major depressive disorder studies				
Caso et al. (2021) (50) Cross sectional study design *(Web of Science, MEDLINE, Scopus)*	Identify whether human faecal microbiota is altered in MDD vs. HC	16 s rDNA (V3-V4 region); QIIME pipeline analysis (v1.8.0)	Patients with MDD Active MDD (*n* = 46); MDD in remission or mild MDD (*n* = 22); HC (*n* = 46)	MDD vs. HC Increased abundance: *Bilophila* (2-fold), *Alistipes* (1.5-fold) Decreased abundance: *Anaerostipes* (1.5-fold), *Dialister* (15-fold)
Huang et al. (2018) (51) Cross sectional study design *(Hand searched literature)*	Define the shifts of Firmicutes in MDD	16 s rRNA (V3-V4 region); QIIME pipeline analysis (v1.9.1)	Han Chinese patients with MDD MDD patients (*n* = 27); HC (*n* = 27)	MDD vs. HC (reported with LDA score (log 10) > 2.0) Increased abundance: *Oxalobacter, Pseudomonas, Parvimonas, Bulleidia, Peptostreptococcus, Gemella* Decreased abundance: *Firmicutes, Lachnospiraceae, Ruminococcaceae, Clostridiaceae* *Coprococcus, Blautia, Dorea*
Liu et al. (2022) (52) Cross sectional study design *(MEDLINE, Scopus)*	Analyze the gut microbiota composition in MDD patients	16 s rRNA; QIIME pipeline analysis (v 2019.1)	First-Episode MDD inpatients First-Episode MDD patients (*n* = 66); HC (*n* = 43)	MDD vs. HC (reported with LDA score (log2), *p* < 0.05) Increased abundance: *Deinococcaceae* *Deinococcus, Odoribacter* Decreased abundance: *Bacteroidaceae, Turicibacteraceae, Clostridiaceae, Barnesiellaceae* *Alistipes, Turicbacter, Clostridium, Roseburia, Enterobacter, Bacteroides*
Sun et al. (2022) (53) Cross sectional study design *(Web of Science, MEDLINE, Scopus, Academic Search Ultimate)*	Measure correlation between gut microbiota and MDD	16 s rRNA (V3-V4 region); QIIME2 pipeline analysis	Patients with MDD MDD patients (*n* = 31); HC (*n* = 29)	MDD vs. HC (reported with LDA score (log 10) > 2.0) Increased abundance: *Deinococcales Deinococcaceae Deinococcus* Decreased abundance: *Bacteroidetes Bacteroidia Bacteroidales, Turicibacterales Bacteroidaceae, Clostridiaceae, Barnesielllaceae, Turicibactereaceae Bacteriodes, Ruminococcus, Clostridium, Barnesiella, Turicibacter*
Low fermentable oligosaccharide, disaccharide, monosaccharide & polyol studies				
McIntosh et al. (2017) (54) Controlled, single blind randomised study Duration: 21 days *(Web of Science, MEDLINE, Scopus, Cochrane)*	Comparison of the impact of a low FODMAP diet vs. high FODMAP diet on the microbiome of patients with IBS	16S rRNA (V3 region); QIIME pipeline analysis	IBS patients (Rome III criteria) Low FODMAP diet (*n* = 19); High FODMAP diet (*n* = 18)	Low vs. High FODMAP diet Increased abundance: *Firmicutes* (*p* = 0.029)*, Actinobacteria* (*p* = 0.029), *Clostridiales*, (*p* = 0.023), *Clostridiales family XIII Incertae sedis* (*p* = 0.008) *Porphyromonas* (*p* = 0.01) Decreased abundance: *Propionibacteriaceae* (*p* = 0.043)
Staudacher et al. (2017) (55) Randomized, placebo-controlled study Duration: 4 weeks *(MEDLINE, Scopus, Cochrane)*	Determine effect of low FODMAP diet +/− probiotic on faecal microbiota in patients with IBS	16 s rRNA (V3-V4 region); QIIME v1.9	IBS patients (Rome III criteria) from the United Kingdom Sham diet/placebo (*n* = 27); Low FODMAP diet/placebo (*n* = 24)	Low FODMAP diet/placebo v sham diet/placebo Abundance decreased: *Bifidobacterium* (mean difference-0.39 rRNA genes/g, 95% CI -0.64 to −0.13, P + 0.008)
Staudacher et al. (2021) (56) 2×2 factorial design randomized controlled trial Duration: 4 weeks *(Web of Science, MEDLINE, Scopus, Cochrane)*	Identify diet-microbiota associations in adults with IBS consuming a habitual diet and the impact of consuming two nutritional interventions for IBS	16 s rRNA (V3-V4 region); QIIME v1.9	IBS patients (Rome III criteria) from the United Kingdom Sham diet/placebo (*n* = 24); Low FODMAP diet/placebo (*n* = 21)	Low FODMAP diet/placebo v sham diet/placebo Abundance increased: *Bacteroidetes* (q = 0.05)*, Bacteroidaceae* (q = 0.008)*, Bacteriodes* (34.1% (15.7%) vs. 23.3% (15.2%), q = 0.01) Abundance decreased: *Actinobacteria* (q = 0.007), *Firmicutes* (q + 0.05)*, Bifidobacteriaceae* (q = 0.016)*, Ruminococcaceae* (8.3% (5.1%) vs. 12.8% (5.9%), q = <0.001)*, Bifidobacterium* (0.9% (1.0%) vs. 2.1% (2.5%), q = 0.029)
Zhang et al. (2021) (57) Randomized, parallel-group controlled study Duration: 3 weeks *(Cochrane)*	Determine the efficacy and factors of a low FODMAP diet compared to TDA	16 s rRNA (V3-V4 region)	Chinese IBS – diarrhoea (Rome III criteria) patients Low FODMAP diet (*n* = 30); TDA (*n* = 26)	Low FODMAP vs. TDA (reported with LDA score (log 10) > 2.0) Abundance increased: *Bilophila* Abundance decreased: *Actinobacteria, Bifidobacterium, Fusobacterium*

MDD, major depressive disorder; HC, healthy controls; FODMAP, fermentable, oligosaccharides, disaccharides, monosaccharide, and polyols; IBS, irritable bowel syndrome; TDA, traditional dietary advice.

## 3 Results

**Figure 1:**
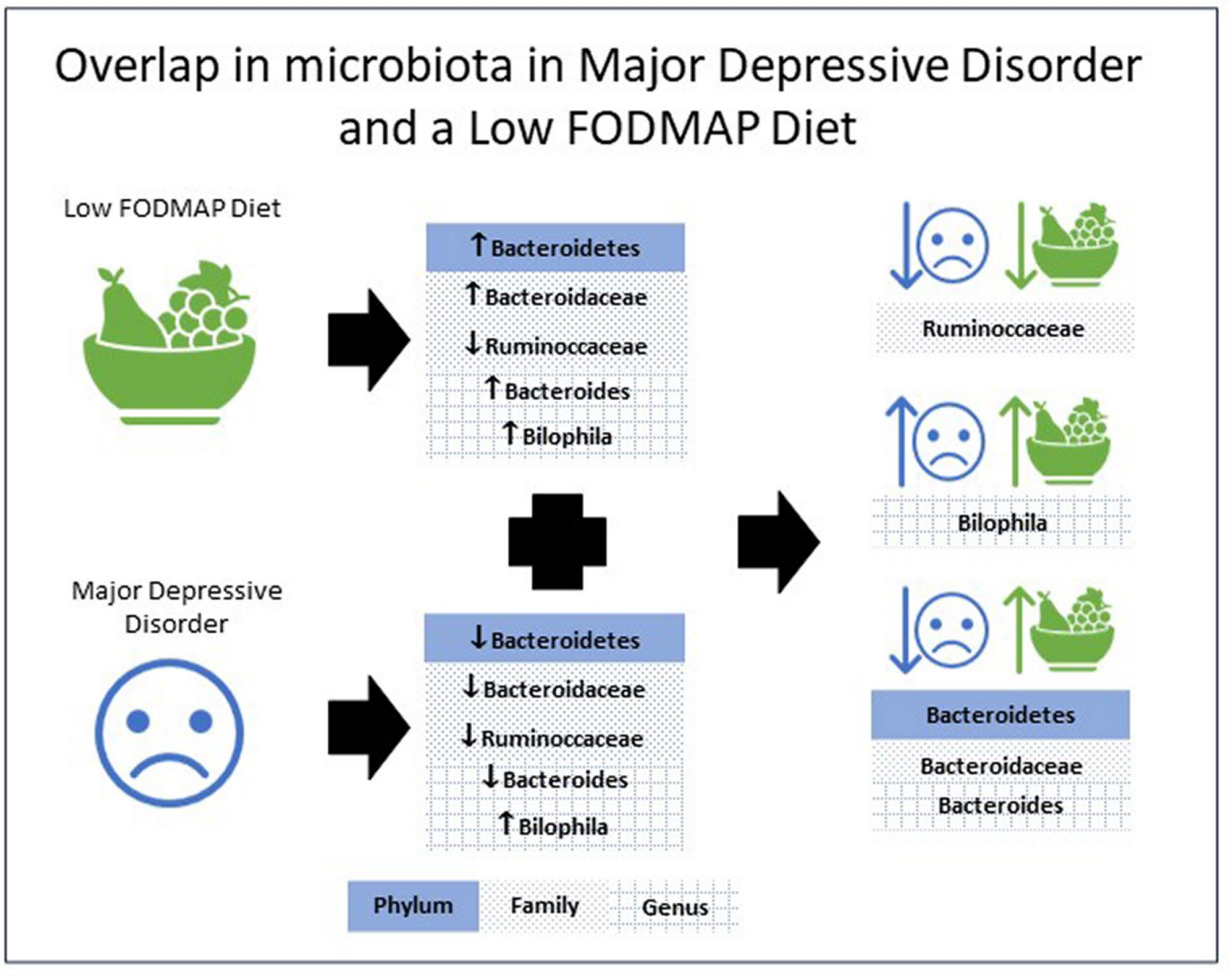
Overlap in the alterations in microbiota abundance in major depressive disorder and with a low FODMAP diet.

Table 1 presents research examining the gut microbiota in individuals with MDD, as well as the affect FODMAP or habitual diets have on the gut microbiota. Analysis of the eight included studies indicate the phylum *Bacteroidetes* (53, 56), the family *Bacteroidaceae* (52, 53, 56) and the genus *Bacteroides* (52, 53, 56) all increased with a low FODMAP diet and were lower with MDD. The family *Ruminoccaceae* (51, 56) were lower with a low FODMAP diet and MDD, while the genus *Bilophila* (50, 57) were higher with a low FODMAP diet and MDD. For all other microbiota, results were inconsistent, e.g., *Actinobacteria* (54, 56, 57), *Firmicutes* (54, 56) either increased or decreased with the low FODMAP diet, and *Alistipes* (50, 52) was higher or lower with MDD, or did not overlap between MDD and the low FODMAP diet. Figure 1 details the overlap in alterations in gut microbiota in MDD and low FODMAP intake.

## 4 Discussion

The overlap in microbiota shifts revealed in this review provide preliminary evidence of a potential mechanism for a low FODMAP diet to benefit MDD (21, 22). Overall, the data shows that using a low FODMAP diet can drive the microbiota in a manner that may benefit MDD. This outcome provides exploratory justification to evaluate a low FODMAP diet as an adjunctive treatment for MDD.

In agreement with the recent review by Knuesel and Mohajeri (25) the studies reported higher prevalence of *Coprococcus* (LDA > 2) (51), *Roseburia* (LDA > 2) (52), and *Ruminococcaceae* (LDA > 2) (51) in healthy controls compared to MDD patients. However, the studies also detailed alterations to the gut microbiota that contradict earlier findings including a lower abundance of *Bacteroides* (LDA > 2) (53) which were previously reported to increase with MDD. Moreover, *Alistipes* (50, 52) was higher or lower depending on the study considered, whereas the earlier review concluded that *Alistipes* were more abundant in MDD subjects (25). These differences from the earlier review may be attributed to the broader selection criteria with no consideration given to medication intake, or the bioanalytical pipelines used.

A limitation of the MDD and gut microbiota studies is the lack of consideration for symptom severity at the time of data collection. Evidence shows that changes in symptom severity are linked to changes within the microbiota with small increases in *Phascolarbacterium* and *Akkermansia* associated with elevated depressive symptomology (59). Furthermore Huang et al. (51) focused on a single phylum, *Firmicutes*, limiting data for the complete gut microbiota. Another limitation of the studies investigating MDD, and the microbiota is the lack of consideration of diet quality. Epidemiological data shows an inverse relationship between diet quality and MDD (10) and diet quality is known to impact on the microbiota (60). To better understand the differences between individuals living with MDD and the control group, it would be beneficial to detail diet quality in both clinical and research settings.

Studies examining changes in the gut microbiota when a low FODMAP diet is consumed produced results that align with expected changes in macronutrient profile with a low FODMAP diet. Trials examining macronutrient intake during a low FODMAP diet show that carbohydrates decreased, while protein and fat remained the same as dietary controls (61). Therefore, changes to the microbiota associated with increased fat and protein intake might be expected. An increased abundance of *Firmicutes* (54) *Clostridales* (54), *Bacteroides* (56), and *Bilophila* (57) are all associated with increased total dietary fat intake. A low FODMAP intake yielded an increase in *Bacteroidetes* (56), with this phylum associated with decreased abundance when dietary intake was high in saturated fats, indicating that the dietary fat intake may be skewed toward the healthier unsaturated fats. An increase is seen in the prevalence of *Bacteroides* (55) and *Bilophila* (57), both of which are associated with increased protein intake and a decrease in *Bifidobacterium* (55–57). *Bifidobacterium* is increased with consumption of plant-based proteins such as legumes which typically reduce in a low FODMAP diet given their galacto-oligosaccharide content.

The reduction in carbohydrate commonly found in a low FODMAP diet has implications for the type and quantity of fiber intake (61). Low FODMAP diets reduce intake of fructose in excess of glucose (62) which partially explains the reduction in *Bifidobacterium* (55) and increase in *Bacteroides* (56) seen in low FODMAP studies. A reduction in fiber intake is common when undertaking a low FODMAP elimination diet, unless a concerted effort is made to replace the relinquished foods with suitable alternatives (63). Study outcomes align with the anticipated outcomes associated with a reduction in fiber intake, with an increase in *Bilophila* (57), *Bacteroidetes* (56) and *Firmicutes* (54) bacteria that decrease with fiber consumption. A decrease in the abundance of *Bifidobacteria* (55) which increase with fiber consumption, was evident. No change in *Lactobacilli* (55) was observed which increase with inulin and resistant starch intake, but an increase in *Actinobacteria* (54) that increase with consumption of resistant starch was evident.

A limitation of the low FODMAP studies is the lack of clarity on whether the dietetic intervention was provided by qualified clinicians. Dietary outcomes improve when delivery is conducted by dietitians (64). All the studies require more accurate measures of dietary compliance (65). Two studies used self-disclosure by the participants (55, 56), while the other two studies did not include any dietary compliance measure (54, 57). Biomarkers can provide additional confirmation of dietary intake to support self-reported assessments (66).

A potential limitation is the higher abundance of *Bilophila* (50, 57) and lower abundance of *Ruminoccaccaceae* (51, 56) in both MDD and the low FODMAP. However, with carefully targeted dietary counselling these negative changes may possibly be offset. For example, nut consumption purportedly increases the abundance of *Ruminococcaceae* (67, 68) and could easily be promoted within a low FODMAP diet. Increased *Bilophila* is associated with high consumption of animal fats (69) thus counselling to reduce intake may help in resolving the increased *Bilophila* reported in both MDD and low FODMAP elimination diets. An overall improvement to diet quality could further offset some of these negative impacts (60).

Given that poor diet quality is associated with MDD (70), specific changes that focus on improving diet quality should be considered as an adjunctive treatment to improve outcomes for individuals living with MDD. Diet quality has also been associated with microbiota composition. For example, higher quality diets, as measured by the Healthy Eating Index, have a 13%–19% lower relative abundance of *Actinobacteria* (60). Studies investigating diet quality of a low FODMAP elimination diet have reported both quality improvement (71, 72), and a decline in quality in adults (73). A low FODMAP approach is not always undertaken with appropriate dietetic supervision, particularly with adults which has consequences for diet quality an outcome (74). A low FODMAP diet should be supervised by a qualified dietitian with good understanding of the interactions between the microbiota and dietary change to achieve optimal results.

## 5 Conclusion

There is a need to better understand the potential for dietary intervention to impact MDD via modification of the microbiome. To provide evidence-based clinical guidelines, further studies are needed to characterize changes in the microbiota over time rather than at a single time point. Consistent use of bioanalytical pipelines to allow clearer comparisons across data is required. Better methodological controls need to be implemented regarding intervention delivery, and dietary compliance. To increase the validity of comparisons between studies, the severity of MDD experienced and diet quality need to be addressed.

## Author contributions

SO’N: Conceptualization, Investigation, Methodology, Writing – original draft. MM: Conceptualization, Supervision, Writing – review & editing. CK-A: Conceptualization, Supervision, Writing – review & editing. DP: Conceptualization, Supervision, Writing – review & editing.

## References

[1] Marx W Lane M Hockey M Aslam H Berk M Walder K. Diet and depression: exploring the biological mechanisms of action. Mol Psychiatry. (2021) 26:134–50. doi: 10.1038/s41380-020-00925-x, PMID: 33144709

[2] Cotman CW Berchtold NC Christie L-A. Exercise builds brain health: key roles of growth factor cascades and inflammation. Trends Neurosci (Regular ed). (2007) 30:464–72. doi: 10.1016/j.tins.2007.06.011, PMID: 17765329

[3] Eyre H Baune BT. Neuroimmunological effects of physical exercise in depression. Brain Behav Immun. (2011) 26:251–66. doi: 10.1016/j.bbi.2011.09.015, PMID: 21986304

[4] Malhi GS Mann JJ. Depression. Lancet. (2018) 392:2299. doi: 10.1016/S0140-6736(18)31948-2 30396512

[5] Casacalenda N Perry JC Looper K. Remission in major depressive disorder: a comparison of pharmacotherapy, psychotherapy, and control conditions. Am J Psychiatry. (2002) 159:1354–60. doi: 10.1176/appi.ajp.159.8.1354, PMID: 12153828

[6] Machmutow K Meister R Jansen A Kriston L Watzke B Harter MC. Comparative effectiveness of continuation and maintenance treatments for persistent depressive disorder in adults. Cochrane Database Syst Rev. (2019) 5:CD012855. Epub 2019/05/21. doi: 10.1002/14651858.CD012855.pub2, PMID: 31106850 PMC6526465

[7] Hengartner MP. How effective are antidepressants for depression over the Long term? A critical review of relapse prevention trials and the issue of withdrawal confounding. Ther Adv Psychopharma. (2020) 10:2045125320921694. doi: 10.1177/2045125320921694, PMID: 32435449 PMC7225779

[8] Malhi GS Bell E Bassett D Boyce P Bryant R Hazell P. The 2020 Royal Australian and new Zealand College of Psychiatrists Clinical Practice Guidelines for mood disorders. Aust N Z J Psychiatry. (2021) 55:7–117. doi: 10.1177/0004867420979353, PMID: 33353391

[9] Marx W Manger SH Blencowe M Murray G Ho FY-Y Lawn S. Clinical guidelines for the use of lifestyle-based mental health Care in Major Depressive Disorder: world Federation of Societies for biological psychiatry (Wfsbp) and Australasian Society of Lifestyle Medicine (Aslm) taskforce. World J Biol Psychiatry. (2022) 24:333–86. doi: 10.1080/15622975.2022.2112074, PMID: 36202135 PMC10972571

[10] Lassale C Batty GD Baghdadli A Jacka F Sánchez-Villegas A Kivimäki M. Healthy dietary indices and risk of depressive outcomes: a systematic review and Meta-analysis of observational studies. Mol Psychiatry. (2019) 24:965–86. doi: 10.1038/s41380-018-0237-8, PMID: 30254236 PMC6755986

[11] Molendijk M Molero P Ortuño Sánchez-Pedreño F Van der Does W Angel M-GM. Diet quality and depression risk: a systematic review and dose-response Meta-analysis of prospective studies. J Affec Disord. (2018) 226:346–54. doi: 10.1016/j.jad.2017.09.022, PMID: 29031185

[12] Jacka FN O’Neil A Opie R Itsiopoulos C Cotton S Mohebbi M. A randomised controlled trial of dietary improvement for adults with major depression (the ‘Smiles’ trial). BMC Med. (2017) 15:23. doi: 10.1186/s12916-017-0791-y, PMID: 28137247 PMC5282719

[13] Francis HM Stevenson RJ Chambers JR Gupta D Newey B Lim CK. A brief diet intervention can reduce symptoms of depression in young adults—a randomised controlled trial. PLoS One. (2019) 14:e0222768. doi: 10.1371/journal.pone.0222768, PMID: 31596866 PMC6784975

[14] Parletta N Zarnowiecki D Cho J Wilson A Bogomolova S Villani A. A Mediterranean-style dietary intervention supplemented with fish oil improves diet quality and mental health in people with depression: a randomized controlled trial (Helfimed). Nutr Neurosci. (2019) 22:474–87. doi: 10.1080/1028415X.2017.1411320, PMID: 29215971

[15] Bayes J Schloss J Sibbritt D. A randomised controlled trial assessing the effect of a Mediterranean diet on the symptoms of depression in young men (the ‘Ammend’ study): a study protocol. Br J Nutr. (2021) 126:730–7. doi: 10.1017/S0007114520004699, PMID: 33222703

[16] Burrows T Teasdale S Rocks T Whatnall M Schindlmayr J Plain J. Effectiveness of dietary interventions in mental health treatment: a rapid review of reviews. Nutr Diet. (2022) 79:279–90. doi: 10.1111/1747-0080.12754, PMID: 35796181 PMC9545734

[17] Cryan JF O’Riordan KJ Cowan CSM Sandhu KV Bastiaanssen TFS Boehme M. The microbiota-gut-brain Axis. Physiol Rev. (2019) 99:1877–2013. doi: 10.1152/physrev.00018.2018 31460832

[18] McGuinness AJ Davis JA Dawson SL Loughman A Collier F O’Hely M. A systematic review of gut microbiota composition in observational studies of major depressive disorder, Bipolar Disorder and Schizophrenia. Mol Psychiatry. (2022) 27:1920–35. doi: 10.1038/s41380-022-01456-3, PMID: 35194166 PMC9126816

[19] Graf D Di Cagno R Fåk F Flint HJ Nyman M Saarela M. Contribution of diet to the composition of the human gut microbiota. Microb Ecol Health Dis. (2015) 26:26164. doi: 10.3402/mehd.v26.26164, PMID: 25656825 PMC4318938

[20] Ong DK Mitchell SB Barrett JS Shepherd SJ Irving PM Biesiekierski JR. Manipulation of dietary short chain carbohydrates alters the pattern of gas production and genesis of symptoms in irritable bowel syndrome. J Gastroenterol Hepatol. (2010) 25:1366–73. Epub Accepted for publication 26 April 2010. doi: 10.1111/j.1440-1746.2010.06370.x 20659225

[21] Peters SL Yao CK Philpott H Yelland GW Muir JG Gibson PR. Randomised clinical trial: the efficacy of gut-directed hypnotherapy is similar to that of the low Fodmap diet for the treatment of irritable bowel syndrome. Aliment Pharmacol Ther. (2016) 44:447–59. doi: 10.1111/apt.13706, PMID: 27397586

[22] Eswaran S Chey WD Jackson K Pillai S Chey SW Han-Markey T. A diet low in fermentable oligo-, Di-, and monosaccharides and polyols improves quality of life and reduces activity impairment in patients with irritable bowel syndrome and diarrhea. Clin Gastroenterol Hepatol. (2017) 15:1890–9.e3. doi: 10.1016/j.cgh.2017.06.044, PMID: 28668539

[23] Ribeiro G Ferri A Clarke G Cryan JF. Diet and the microbiota–gut–brain-Axis: a primer for clinical nutrition. Curr Opin Clin Nutr Metab Care. (2022) 25:443–50. doi: 10.1097/MCO.0000000000000874, PMID: 36102353 PMC9553262

[24] Liang S Wu X Hu X Wang T Jin F. Recognizing depression from the microbiota^−^Gut^−^Brain Axis. Int J Mol Sci. (2018) 19:1592. doi: 10.3390/ijms19061592, PMID: 29843470 PMC6032096

[25] Knuesel T Mohajeri MH. The role of the gut microbiota in the development and progression of major depressive and bipolar disorder. Nutrients. (2022) 14:37. doi: 10.3390/nu14010037, PMID: 35010912 PMC8746924

[26] Foster JA McVey Neufeld K-A. Gut–brain Axis: how the microbiome influences anxiety and depression. Trends Neurosci. (2013) 36:305–12. doi: 10.1016/j.tins.2013.01.005 23384445

[27] Yu S Wang L Jing X Wang Y An C. Features of gut microbiota and short-chain fatty acids in patients with first-episode depression and their relationship with the clinical symptoms. Front Psychol. (2023) 14:1088268. doi: 10.3389/fpsyg.2023.1088268, PMID: 37168424 PMC10165121

[28] Mirzaei R Bouzari B Hosseini-Fard SR Mazaheri M Ahmadyousefi Y Abdi M. Role of microbiota-derived short-chain fatty acids in nervous system disorders. Biomed Pharmacother. (2021) 139:111661. doi: 10.1016/j.biopha.2021.111661, PMID: 34243604

[29] Hayley S Audet M-C Anisman H. Inflammation and the microbiome: implications for depressive disorders. Curr Opin Pharmacol. (2016) 29:42–6. doi: 10.1016/j.coph.2016.06.001 27327647

[30] Chen J-J Zheng P Liu Y-Y Zhong X-G Wang H-Y Guo Y-J. Sex differences in gut microbiota in patients with major depressive disorder. Neuropsychiatr Dis Treat. (2018) 14:647–55. doi: 10.2147/NDT.S159322, PMID: 29520144 PMC5833751

[31] Chen J-J He S Fang L Wang B Bai S-J Xie J. Age-specific differential changes on gut microbiota composition in patients with major depressive disorder. Aging (Albany NY). (2020) 12:2764–76. doi: 10.18632/aging.102775, PMID: 32040443 PMC7041727

[32] David LA Maurice CF Carmody RN Gootenberg DB Button JE Wolfe BE. Diet rapidly and reproducibly alters the human gut microbiome. Nature. (2014) 505:559–63. doi: 10.1038/nature12820, PMID: 24336217 PMC3957428

[33] Garcia-Mantrana I Selma-Royo M Alcantara C Collado MC. Shifts on gut microbiota associated to Mediterranean diet adherence and specific dietary intakes on general adult population. Front Microbiol. (2018) 9:890. doi: 10.3389/fmicb.2018.00890, PMID: 29867803 PMC5949328

[34] Matijašić BB Obermajer T Lipoglavšek L Grabnar I Avguštin G Rogelj I. Association of Dietary Type with fecal microbiota in vegetarians and omnivores in Slovenia. Eur J Nutr. (2014) 53:1051–64. doi: 10.1007/s00394-013-0607-6, PMID: 24173964

[35] Kabeerdoss J Shobana Devi R Regina Mary R Ramakrishna BS. Faecal microbiota composition in vegetarians: comparison with omnivores in a cohort of young women in southern India. Br J Nutr. (2012) 108:953–7. doi: 10.1017/S0007114511006362 22182464

[36] Singh RK Chang H-W Yan D Lee KM Ucmak D Wong K. Influence of diet on the gut microbiome and implications for human health. J Transl Med. (2017) 15:73. doi: 10.1186/s12967-017-1175-y, PMID: 28388917 PMC5385025

[37] Rinninella E Cintoni M Raoul P Lopetuso LR Scaldaferri F Pulcini G. Food components and dietary habits: keys for a healthy gut microbiota composition. Nutrients. (2019) 11:2393. doi: 10.3390/nu11102393, PMID: 31591348 PMC6835969

[38] Tir Touil Meddah A Yazourh A Desmet I Risbourg B Verstraete W Romond MB. Regulatory effects of whey Retentate from Bifidobacteria fermented Milk on the microbiota of the simulator of the human intestinal microbial ecosystem (Shime). J Appl Microbiol. (2001) 91:1110–7. doi: 10.1046/j.1365-2672.2001.01482.x, PMID: 11851820

[39] Dominika Ś Arjan N Karyn RP Henryk K. The study on the impact of glycated pea proteins on human intestinal Bacteria. Int J Food Microbiol. (2011) 145:267–72. doi: 10.1016/j.ijfoodmicro.2011.01.002 21276631

[40] Kim CH Park J Kim M. Gut microbiota-derived short-chain fatty acids, T cells, and inflammation. Immune Netw. (2014) 14:277–88. doi: 10.4110/in.2014.14.6.277, PMID: 25550694 PMC4275385

[41] Fava F Gitau R Griffin BA Gibson GR Tuohy KM Lovegrove JA. The type and quantity of dietary fat and carbohydrate Alter Faecal microbiome and short-chain fatty acid excretion in a metabolic syndrome ‘at-Risk’ population. Int J Obes. (2013) 37:216–23. doi: 10.1038/ijo.2012.33, PMID: 22410962

[42] Wu GD Chen J Hoffmann C Bittinger K Chen Y-Y Keilbaugh SA. Linking Long-term dietary patterns with gut microbial Enterotypes. Science. (2011) 334:105–8. doi: 10.1126/science.1208344, PMID: 21885731 PMC3368382

[43] Hills JRD Pontefract BA Mishcon HR Black CA Sutton SC Theberge CR. Gut microbiome: profound implications for diet and disease. Nutrients. (2019) 11:1613. doi: 10.3390/nu11071613, PMID: 31315227 PMC6682904

[44] Whitney EN Rady Rolfes S Crowe T Walsh A. Understanding Nutrition. 3 Australian and new Zealand edition. ed. South Melbourne, Vic: Cengage Learning (2017).

[45] Eid N Enani S Walton G Corona G Costabile A Gibson G. The impact of date palm fruits and their component polyphenols, on gut microbial ecology, bacterial metabolites and Colon Cancer cell proliferation. J Nutr Sci (Cambridge). (2014) 3:e46-e. doi: 10.1017/jns.2014.16, PMID: 26101614 PMC4473134

[46] Linetzky Waitzberg D Alves Pereira CC Logullo L Manzoni Jacintho T Almeida D. Teixeira da Silva ML, et al. microbiota benefits after inulin and partially Hydrolized guar gum supplementation: a randomized clinical trial in constipated women. Nutr Hosp. (2012) 27:123–9. doi: 10.1590/S0212-16112012000100014 22566311

[47] Costabile A Kolida S Klinder A Gietl E Bäuerlein M Frohberg C. A double-blind, placebo-controlled, cross-over study to establish the bifidogenic effect of a very-Long-chain inulin extracted from globe artichoke (*Cynara Scolymus*) in healthy human subjects. Br J Nutr. (2010) 104:1007–17. doi: 10.1017/S0007114510001571, PMID: 20591206

[48] Walker AW Ince J Duncan SH Webster LM Holtrop G Ze X. Dominant and diet-responsive groups of Bacteria within the human colonic microbiota. ISME J. (2011) 5:220–30. doi: 10.1038/ismej.2010.118, PMID: 20686513 PMC3105703

[49] Martínez I Kim J Duffy PR Schlegel VL Walter J. Resistant starches types 2 and 4 have differential effects on the composition of the fecal microbiota in human subjects. PLoS One. (2010) 5:e15046. doi: 10.1371/journal.pone.0015046, PMID: 21151493 PMC2993935

[50] Caso JR MacDowell KS González-Pinto A García S de Diego-Adeliño J Carceller-Sindreu M. Gut microbiota, innate immune pathways, and inflammatory control mechanisms in patients with major depressive disorder. Transl Psychiatry. (2021) 11:645. doi: 10.1038/s41398-021-01755-3, PMID: 34934041 PMC8692500

[51] Huang Y Shi X Li Z Shen Y Shi X Wang L. Possible Association of Firmicutes in the gut microbiota of patients with major depressive disorder. Neuropsychiat Dis Treat. (2018) 14:3329–37. doi: 10.2147/NDT.S188340, PMID: 30584306 PMC6284853

[52] Liu P Gao M Liu Z Zhang Y Tu H Lei L. Gut microbiome composition linked to inflammatory factors and cognitive functions in first-episode, drug-naive major depressive disorder patients. Front Neurosci. (2022) 15:800764. doi: 10.3389/fnins.2021.800764, PMID: 35153660 PMC8831735

[53] Sun N Zhang J Wang J Liu Z Wang X Kang P. Abnormal gut microbiota and bile acids in patients with first-episode major depressive disorder and correlation analysis. Psychiatry Clin Neurosci. (2022) 76:321–8. doi: 10.1111/pcn.13368, PMID: 35445772

[54] McIntosh K Reed DE Schneider T Dang F Keshteli AH De Palma G. Fodmaps Alter symptoms and the metabolome of patients with IBS: a randomised controlled trial. Gut. (2017) 66:1241–51. doi: 10.1136/gutjnl-2015-311339, PMID: 26976734

[55] Staudacher HM Lomer MCE Farquharson FM Louis P Fava F Franciosi E. Diet low in Fodmaps reduces symptoms in patients with irritable bowel syndrome and probiotic restores Bifidobacterium species: a randomized controlled trial. Gastroenterology. (2017) 153:936–47. doi: 10.1053/j.gastro.2017.06.010 28625832

[56] Staudacher HM Scholz M Lomer MCE Ralph FS Irving PM Lindsay JO. Gut microbiota associations with diet in irritable bowel syndrome and the effect of low Fodmap diet and probiotics. Clin Nutr. (2021) 40:1861–70. doi: 10.1016/j.clnu.2020.10.013, PMID: 33183883

[57] Zhang Y Feng L Wang X Fox M Luo L Du L. Low fermentable oligosaccharides, disaccharides, monosaccharides, and polyols diet compared with traditional dietary advice for diarrhea-predominant irritable bowel syndrome: a parallel-group, randomized controlled trial with analysis of clinical and microbiological factors associated with patient outcomes. Am J Clin Nutr. (2021) 113:1531–45. doi: 10.1093/ajcn/nqab005, PMID: 33740048

[58] Siegwald L Caboche S Even G Viscogliosi E Audebert C Chabé M. The impact of bioinformatics pipelines on microbiota studies: Does the analytical “microscope” affect the biological interpretation? Microorganisms. (2019) 7:393. doi: 10.3390/microorganisms7100393, PMID: 31561435 PMC6843237

[59] Zhang X Hou Y Li Y Wei W Cai X Shao H. Taxonomic and metabolic signatures of gut microbiota for assessing the severity of depression and anxiety in major depressive disorder patients. Neuroscience. (2022) 496:179–89. doi: 10.1016/j.neuroscience.2022.06.024, PMID: 35750110

[60] Maskarinec G Hullar MAJ Monroe KR Shepherd JA Hunt J Randolph TW. Fecal microbial diversity and structure are associated with diet quality in the multiethnic cohort adiposity phenotype study. J Nutr. (2019) 149:1575–84. doi: 10.1093/jn/nxz065, PMID: 31187868 PMC6862930

[61] Staudacher HM. Nutritional, microbiological and psychosocial implications of the low Fodmap diet. J Gastroenterol Hepatol. (2017) 32:16–9. doi: 10.1111/jgh.13688, PMID: 28244658

[62] Halmos EP Gibson PR. Controversies and reality of the Fodmap diet for patients with irritable bowel syndrome. J Gastroenterol Hepatol. (2019) 34:1134–42. doi: 10.1111/jgh.14650, PMID: 30945376

[63] Tuck CJ Muir JG Barrett JS Gibson PR. Fermentable oligosaccharides, disaccharides, monosaccharides and polyols: role in irritable bowel syndrome. Expert Rev Gastroenterol Hepatol. (2014) 8:819–34. doi: 10.1586/17474124.2014.917956 24830318

[64] Holmes AL Sanderson B Maisiak R Brown A Bittner V. Dietitian services are associated with improved patient outcomes and the Medficts dietary assessment questionnaire is a suitable outcome measure in cardiac rehabilitation. J Am Diet Assoc. (2005) 105:1533–40. doi: 10.1016/j.jada.2005.08.001, PMID: 16183352

[65] Jacka FN. Nutritional psychiatry: where to next? EBioMedicine. (2017) 17:24–9. doi: 10.1016/j.ebiom.2017.02.020, PMID: 28242200 PMC5360575

[66] Trabulsi J Schoeller DA. Evaluation of dietary assessment instruments against doubly labeled water, a biomarker of habitual energy intake. Am J Physiol Endocrinol Metab. (2001) 281:E891–9. doi: 10.1152/ajpendo.2001.281.5.E891 11595643

[67] Sapp PA Kris-Etherton PM Arnesen EA Chen See JR Lamendella R Petersen KS. Peanuts as a nighttime snack enrich butyrate-producing Bacteria compared to an Isocaloric lower-fat higher-carbohydrate snack in adults with elevated fasting glucose: a randomized crossover trial. Clin Nutr (Edinburgh, Scotland). (2022) 41:2169–77. doi: 10.1016/j.clnu.2022.08.004 36067589

[68] Bamberger C Rossmeier A Lechner K Wu L Waldmann E Fischer S. A walnut-enriched diet affects gut microbiome in healthy Caucasian subjects: a randomized, controlled trial. Nutrients. (2018) 10:244. doi: 10.3390/nu10020244, PMID: 29470389 PMC5852820

[69] Wu Y-T Shen S-J Liao K-F Huang C-Y. Dietary plant and animal protein sources oppositely modulate fecal Bilophila and Lachnoclostridium in vegetarians and omnivores. Microbiol Spectr. (2022) 10:e0204721-e. doi: 10.1128/spectrum.02047-21, PMID: 35285706 PMC9045121

[70] Firth J Marx W Dash S Carney R Teasdale SB Solmi M. The effects of dietary improvement on symptoms of depression and anxiety: a Meta-analysis of randomized controlled trials. Psychosom Med. (2019) 81:265–80. doi: 10.1097/PSY.0000000000000673, PMID: 30720698 PMC6455094

[71] Narayana V McMeans AR Levy RL Shulman RJ Chumpitazi BP. Children with functional abdominal pain disorders successfully decrease Fodmap food intake on a low Fodmap diet with modest improvements in nutritional intake and diet quality. Neurogastroenterol Motil. (2022) 34:e14392-n/a. doi: 10.1111/nmo.14392, PMID: 35535019 PMC9529764

[72] Narayana V McMeans AR Levy RL Shulman RJ Chumpitazi BP. Su612 low Fodmap diet adherence is high and positively impacts nutritional intake and diet quality in children with irritable bowel syndrome (Ibs). Gastroenterology. (2021) 160:S-754–5. doi: 10.1016/S0016-5085(21)02515-4

[73] Staudacher HM Ralph FSE Irving PM Whelan K Lomer MCE. Nutrient intake, diet quality, and diet diversity in irritable bowel syndrome and the impact of the low Fodmap diet. J Acad Nutr Diet. (2020) 120:535–47. doi: 10.1016/j.jand.2019.01.017, PMID: 31029650

[74] Van Ouytsel P Szalai A Van Gossum A Arvanitakis M Louis H. Feasibility of a low Fodmaps diet without initial dietician intervention in the Management of Patients with irritable bowel syndrome: a prospective study. Acta Gastro-Enterol. (2021) 84:593–600. doi: 10.51821/84.4.010, PMID: 34965041

